# Human umbilical cord mesenchymal stromal cells exhibit immature nucleus pulposus cell phenotype in a laminin-rich pseudo-three-dimensional culture system

**DOI:** 10.1186/scrt331

**Published:** 2013-10-02

**Authors:** Brian H Chon, Esther J Lee, Liufang Jing, Lori A Setton, Jun Chen

**Affiliations:** 1 Department of Biomedical Engineering, Duke University, Durham, NC 27708, USA; 2 Department of Orthopaedic Surgery, Duke University Medical Center, Box 3093, 375 Medical Science Research Building, Durham, NC 27710, USA

## Abstract

**Introduction:**

Cell supplementation to the herniated or degenerated intervertebral disc (IVD) is a potential strategy to promote tissue regeneration and slow disc pathology. Human umbilical cord mesenchymal stromal cells (HUCMSCs) – originating from the Wharton’s jelly – remain an attractive candidate for such endeavors with their ability to differentiate into multiple lineages. Previously, mesenchymal stem cells (MSCs) have been studied as a potential source for disc tissue regeneration. However, no studies have demonstrated that MSCs can regenerate matrix with unique characteristics matching that of immature nucleus pulposus (NP) tissues of the IVD. In our prior work, immature NP cells were found to express specific laminin isoforms and laminin-binding receptors that may serve as phenotypic markers for evaluating MSC differentiation to NP-like cells. The goal of this study is to evaluate these markers and matrix synthesis for HUCMSCs cultured in a laminin-rich pseudo-three-dimensional culture system.

**Methods:**

HUCMSCs were seeded on top of Transwell inserts pre-coated with Matrigel™, which contained mainly laminin-111. Cells were cultured under hypoxia environment with three differentiation conditions: NP differentiation media (containing 2.5% Matrigel™ solution to provide for a pseudo-three-dimensional laminin culture system) with no serum, or the same media supplemented with either insulin-like growth factor-1 (IGF-1) or transforming growth factor-β1 (TGF-β1). Cell clustering behavior, matrix production and the expression of NP-specific laminin and laminin-receptors were evaluated at days 1, 7, 13 and 21 of culture.

**Results:**

Data show that a pseudo-three-dimensional culture condition (laminin-1 rich) promoted HUCMSC differentiation under no serum conditions. Starting at day 1, HUCMSCs demonstrated a cell clustering morphology similar to that of immature NP cells *in situ* and that observed for primary immature NP cells within the similar laminin-rich culture system (prior study). Differentiated HUCMSCs under all conditions were found to contain glycosaminoglycan, expressed extracellular matrix proteins of collagen II and laminin α5, and laminin receptors (integrin α3 and β4 subunits). However, neither growth factor treatment generated distinct differences in NP-like phenotype for HUCMSC as compared with no-serum conditions.

**Conclusions:**

HUCMSCs have the potential to differentiate into cells sharing features with immature NP cells in a laminin-rich culture environment and may be useful for IVD cellular therapy.

## Introduction

The nucleus pulposus (NP) of the intervertebral disc (IVD) consists of a soft proteoglycan-rich gel possessing high fluid retention capacity. With aging, the NP becomes increasingly dehydrated and may displace or extrude from the IVD due to material failure [1]. This degeneration and associated pathology may manifest back or leg pain or other spine-related disorders [2]. With a very low cell density that decreases with age, the NP itself is not readily capable of self-repair. Furthermore, current treatment options provide only temporary pain relief and may even require invasive procedures [3]. In response to this challenge, cell supplementation to the herniated or degenerated IVD has been explored as a means for stimulating tissue regeneration and hampering disc pathology. In the past decades, many cell sources including autologous chondrocytes and primary IVD cells have been evaluated in clinical or preclinical trials for supplements to promote IVD tissue regeneration [4-6]. However, few sources of healthy autologous cells have been identified except only a small quantity of NP progenitor cells recently confirmed in both young and aged NP tissues [7].

Alternatively, mesenchymal stem cells (MSCs) have been explored as a potential cell source for IVD tissue regeneration [8]. In general, MSCs are capable of differentiating into various cell lineages such as cartilage, bone, adipose tissue and muscle [9,10], and have also shown a potential for reconstructive therapy in orthopedics [11,12]. In the studies of animal models, the transplanting of bone marrow MSCs within hyaluronan gel into rat IVDs and the implantation of atelocollagen-enveloped MSCs into a rabbit IVDs were found to correlate with an increase in disc height and matrix production as compared with no-MSC gels only [13-15]. Recently, MSCs transplanted with fibrous gelatin-transforming growth factor (TGF)-β1 were found to impede apoptosis, thereby maintaining NP cell numbers in the rat IVDs [16]. Similarly, bone marrow MSCs may differentiate into NP-like or chondrocyte-like cells *in vitro*, in alginate three-dimensional culture or in contact with chitosan hydrogels [17-19]. In addition, soluble factors released by NP cells or tissues were found to promote bone marrow MSC differentiation into NP-like cells *in vitro* using condition medium [20,21] and co-culture methods [22,23]. These aforementioned studies show the importance of interactions between bone marrow-derived MSCs cells and the microenvironment for regulating the NP-like phenotype.

Although MSCs from bone marrow source are plentiful, the means of extraction prove somewhat invasive. There is a great clinical advantage to use MSCs derived from the source of waste tissue. It is now known that sufficient quantities of human MSCs bearing multilineage potential can also be obtained from the Wharton’s jelly of the umbilical cord [24], which is usually discarded after birth. Human umbilical cord mesenchymal stromal cells (HUCMSCs) – originating from Wharton’s jelly – are relatively easy to cultivate *in vitro*, have properties that are similar to bone marrow MSCs, express many typical MSC markers (that is, CD29, CD44, CD73, CD90, CD105, CD166, HLA-1) [24], and can differentiate into multiple cell types, such as fibrocartilage [25], cartilage [26], bone [27], neuron [28] and muscle [29]. HUCMSCs also have another advantage of eliciting less immune response during allogeneic transplantation [30]. Of relevance to IVD cell regeneration, HUCMSCs were already found to differentiate into NP-like cells with higher collagen gene expression when co-cultured with human NP cells *in vitro*[31]. There still remains a great need, however, to demonstrate the capability of HUCMSCs to regenerate matrix bearing the unique qualities, the unique cell morphology, and the expression of molecular phenotype/marker matching that of immature NP native tissue.

Immature NP cells originally derived from notochord display distinct morphologic and molecular phenotypes. Notochordal-like NP cells are larger, with vacuoles, abundant cytoplasmic filaments and higher fluorescence as compared with anulus fibrosus cells [32-34]. Several protein markers of NP cells have been suggested, including higher expression of cytokeratin-8 and cytokeratin-19, vimentin, N-cadherin, brachyury, HIF-1α, glucose transporter-1, matrix metalloproteinase-2, CD24, CD44, CD56, galectin-1, type II collagen and aggrecan in NP cells as compared with the neighboring anulus fibrosus cells in IVD tissues [35-42]. Particularly, our group has further discovered that immature NP cells reside in a unique extracellular matrix (ECM) environment rich in laminin and that they express high levels of laminin-binding receptors as compared with the surrounding anulus fibrosus cells [43-45]. In general, immature NP cells (rat, porcine and human) expressed higher levels of integrin subunits α1, α6, β1 and CD239, and higher levels of specific laminin isoforms, LM-511 and LM-322 [44-46]. Functionally, immature porcine NP cells were also shown to interact with laminins predominantly through α6 and β1 integrin subunits [44], and spread significantly more on and adhered with greater strength to laminins (LM-511 and LM-111) as compared with collagen II or fibronectin [46]. Most importantly, both laminin ligand and substrate stiffness modulates immature porcine NP cell clustering behavior on laminin-functionalized gels [47]. Together, our previous findings not only suggest that laminin-rich and soft substrate culture conditions may mimic an immature NP native tissue environment and promote stem cell differentiation to NP-like cells, but also indicate that laminin and its receptors may be useful markers for distinguishing NP-like matrix from cell regeneration. In addition, the cell clustering behavior of NP cells may be a useful feature for distinguishing the NP-like cell phenotype during cell regeneration. In this study, we report that HUCMSCs maintained in a laminin-rich pseudo-three-dimensional culture system were found to exhibit immature NP-like cell phenotype (cell clustering and expressing laminin receptors) and express unique matrix proteins (type II collagen and a specific laminin isoform, LM-511) to NP cells.

## Materials and methods

### Cell culture and flow cytometry analysis

HUCMSCs (passage 2 cells; ScienCell Research Laboratories, Carlsbad, CA, USA) from the Wharton’s jelly of umbilical cords were seeded (5,000 cells/cm^2^) and allowed to proliferate in MSC medium (ScienCell) with 5% fetal bovine serum (ScienCell) and MSC growth supplement (ScienCell) at 37°C and 21% oxygen. All of the following experiments used the same batch of cells, which were isolated from pooled multiple donors (>3) according to the manufacturer’s instructions. Cells were cultured to passage 3 or passage 5. At confluence, passage 3 and passage 5 cells were detached from the culture surface using 0.025% trypsin/ethylenediaminetetraacetic acid (Cambrex, East Rutherford, NJ, USA) and resuspended in culture medium prior to antibody labeling. Cells ((0.25 to 0.5) × 10^6^) were incubated with monoclonal antibodies against CD31, CD34, CD44, CD45, CD90, CD105, CD166, Lutheran glycoprotein (CD239; AbD Serotec, Oxford, UK), integrin subunits α3 (Chemicon, Temecula, CA, USA), β1 (Beckman Coulter, Fullerton, CA, USA), α6 and β4 (BD Pharmingen, San Diego, CA, USA) with appropriate isotype controls and fluorescently labeled secondary antibodies (Millipore, Billerica, MA, USA). Cells were analyzed on a FACScan flow cytometer (Becton Dickinson, Franklin Lakes, NJ, USA) to determine the percentage of cells with positive surface proteins and the mean fluorescence intensity. Flow cytometry analysis was repeated for two sets of cell preparations. The average values for percentage of positive cells and mean fluorescence intensity were reported.

### Differentiation in a laminin-rich pseudo-three-dimensional culture system *in vitro*

HUCMSCs were allowed to differentiate in a laminin-rich culture substrate previously shown to promote the formation of three-dimensional cell clusters for primary NP cells [47]. Then 60 μl of 100% Matrigel™ (growth factor reduced basement membrane matrix containing mainly LM-111; BD Biosciences, San Jose, CA, USA) was coated onto 6.5 mm diameter Costar® Transwell inserts (~0.33 cm^2^ surface/insert for each well of a 24-well culture plate; Corning Incorporated Life Science, Lowell, MA, USA), and allowed to set at 37°C for 2 hours. HUCMSCs at passage 3 were trypsinized and seeded (3 × 10^6^/cm^2^, *n* = 10 wells) on top of Matrigel™ in the Transwell inserts, and then cultured in the following three conditions: with serum-free differentiation media (control = Dulbecco’s modified Eagle’s medium/Ham’s F-12 nutrient mixture + insulin–transferrin–sodium selenite, 50 μg/ml ascorbic acid, 100 nM dexamethasone and 1% nonessential amino acids; all Life Technologies, Grand Island, NY, USA) containing 2.5% Matrigel™ solution (LM-111-rich matrix protein provided a pseudo-three-dimensional condition adapted from previous study for epithelial cell culture [48] in order to promote more cell–laminin interactions during differentiation); under identical conditions supplemented with insulin-like growth factor-1 (IGF-1, 500 ng/ml; courtesy of Dr Masuda); or under identical conditions supplemented with transforming growth factor-β1 (TGF-β1, 1 ng/ml; R&D Systems, Minneapolis, MN, USA). Cells in all three groups were maintained at 37°C under low oxygen tension (2%) conditions in a hypoxia chamber (a modulator incubator chamber flushed with the hypoxic gas as described previously [49]) for 1, 7, 13 and 21 days, and the culture medium was changed with pre-equilibrated new medium (in the same hypoxia chamber for 24 hours) every other day. We choose a hypoxic condition for HUCMSC differentiation here because native NP tissue is a hypoxic environment and hypoxic conditions are an important environmental factor to promote differentiation of MSCs into IVD-like cells, as indicated in a previous study [19]. Following the conclusion of each time point, cells were harvested for histological evaluation or immunostaining (*n* = 3 wells for days 1, 7 and 21), RNA isolation (*n* = 3 wells for days 1 and 7) and biochemistry analysis (*n* = 4 wells for days 0, 7, 13 and 21), as described below.

### Histological evaluation for cell morphology and proteoglycan composition

At the end of each time point, cell samples were collected from the surface of Matrigel™ and embedded in optimum cutting temperature medium (Sakura Finetek, Torrance, CA, USA) and immediately flash-frozen in liquid nitrogen. They were subsequently stored at -80°C until cryosectioning. Frozen tissue sections (7 μm) were then fixed in 10% neutral buffered formalin (Azer Scientific, Morgantown, PA, USA) for 10 minutes, and washed in 1% lithium carbonate solution (Mallinckrodt Chemicals, Phillipsburg, NJ, USA) and stained with 0.5% safranin-O solution (Sigma, St Louis, MO, USA) for 60 seconds. Samples were rinsed with distilled water and counterstained with Mayer’s hematoxylin (Sigma) to visualize individual cells. After serial steps of dehydration, sections were then mounted with Permount (Fisher Scientific, Pittsburg, PA, USA) and visualized with light microscopy for intensity of glycosaminoglycan staining.

### Immunohistochemical staining for type II collagen

Frozen sections were processed for immunohistochemical labeling of type II collagen (HistoStain plus broad spectrum staining kit; Invitrogen, Camarillo, CA, USA) as per the manufacturer’s instructions. Following peroxide blocking, sections were digested with pepsin (Digest-All 3; Invitrogen) for 20 minutes at 37°C. This enzyme helped expose the type II collagen epitope recognized by the mouse monoclonal anti-chicken antibody (II-II6B3; Developmental Studies Hybridoma Bank, Iowa City, IA, USA). Following phosphate-buffered saline washing, sections were incubated with the following reagents provided in the kit: biotinylated secondary antibody (30 minutes), horseradish peroxidase-conjugated streptavidin (30 minutes) and substrate–chromagen mixture (3-amino-9-ethylcarbazole solution; Invitrogen, 10 minutes), which results in a deep red color for positive staining and visualizing the location of the antigen. Finally, sections were counterstained with Mayer’s Hematoxylin to visualize individual cells and mounted in GVA mounting medium (Sigma) for light microscopy. Negative control sections were processed in parallel with mouse IgG isotype control (Millipore) instead of the primary antibody.

### Immunohistochemical staining for immature nucleus pulposus markers

Based on our previous molecular phenotype study of immature NP cells cross three species (rat, porcine and human) [45], laminin α5 and its specific receptor, Lutheran glycoprotein (CD239), other receptors for integrin subunits α3, α6, β4 and β1 were selected here as immature NP markers for evaluation of immature NP-like cell phenotype after HUSMC differentiation. Frozen tissue sections were fixed in 4% formaldehyde (10 minutes at room temperature) for labeling with antibodies detecting the laminin α5 subunit and CD239. For labeling of integrin subunits, tissue sections were fixed in acetone (10 minutes at -20°C). The fixed samples were incubated with a blocking solution (3.75% bovine serum albumin/5% goat serum; Invitrogen) for 30 minutes, and then incubated for 2 hours with one of the following antibodies: monoclonal or polyclonal anti-human laminin α5 chain, CD239, and integrin subunits α3, α6, β4 and β1 (Table 1). Sections were washed twice in phosphate-buffered saline and incubated with appropriate secondary antibodies (AlexaFluor 488 or 633 secondary antibodies; Molecular Probes, Eugene, OR, USA) for 30 minutes in blocking solution. Negative control sections were incubated with appropriate isotype IgG controls (mouse IgG1, IgG2a or rat IgG2a; Millipore) instead of primary antibody, or with secondary antibody alone as a negative control (polyclonal antibodies). All sections were counterstained with propidium iodide (0.2 mg/ml; Sigma) to label cell nuclei, and were imaged using confocal laser scanning microscopy (Zeiss LSM 510; 20× NA 0.5 and 63× water immersion NA 1.2 objectives; Carl Zeiss, Thornwood, NY, USA).

**Table 1:** PCR Probes and primers of nucleus pulposus markers and corresponding antibodies for immunostaining for protein expression

**Target**	**PCR probe/primers**	**Antibody**	
	**Order number**	**Vendor (order number)**	**Host/type**
Aggrecan	Hs 00153936_m1	NA	NA
Type II collagen	Hs 00156568_m1	Developmental Studies Hybridoma Bank, University of Iowa, Iowa City, IA, USA (II-II6B3)	Ms/monoclonal
Integrin α3	Hs 01076873_m1	Chemicon, Temecula, CA, USA (AB1920)	Rb/polyclonal
Integrin α6	Hs 00173952_m1	BD Biosciences, San Jose, CA, USA (555734)	Rat/monoclonal
Integrin β4	Hs 00173995_m1	Chemicon (AB1922)	Rb/polyclonal
CD239 (Lu)	Hs 00170663_m1	AbD Serotec, Oxford, UK (MCA1982)	Ms/monoclonal
Laminin α5	NA	Chemicon (MAB1924)	Ms/monoclonal

Probes and primers from Applied Biosystems (Foster City, CA, USA) of nucleus pulposus markers analyzed by real-time PCR and corresponding antibodies used for immunostaining for protein expression of these markers. Rb, rabbit; Ms, mouse; NA, not applicable; Lu, Lutheran glycoprotein.

### RNA isolation and real-time RT-PCR

Total RNA was extracted from cells in each group (control, TGF-β1 and IGF-1 treatments, *n* = 4) at the end of each culture time point (1 and 7 days) with the RNAeasy kit plus DNase I digestion (Qiagen, Valencia, CA, USA), as described previously [50]. For quantitation of mRNA, each target gene had two human-specific PCR primers and one fluorescently labeled intron-spinning probe (Applied Biosystems, Foster City, CA, USA; Table 1). Amplification conditions were as described previously [50]. Relative gene expression differences were quantified amongst the control and treatment groups at different time points using the comparative C_t_ method with β2-microglobulin as an internal control. The relative mRNA level of the control sample at day 1 was set as the calibrator (value = 1), and all treatment groups at day 1 and all groups at day 7 were normalized by this value. Duplicate PCR reactions were performed for each target gene and the internal control for one RNA sample. Statistical analyses were performed to detect a difference in ∆C_t_ values (C_t_ of target – C_t_ of β2-microglobulin) between day 1 and day 7 samples in each treatment group using a one-factor analysis of variance (ANOVA) (StatView; SAS Institute, Cary, NC, USA) followed by Tukey’s *post-hoc* analysis at a significant level of 0.05 (*n* = 3). Fold-differences of relative mRNA level (2^–∆∆Ct^) between day 1 and day 7 samples in each treatment group were reported if ≥2, and where ANOVA detected a difference at *P* < 0.05 [50].

### Sulfated glycosaminoglycan production

Differentiated HUCMSC production of sulfated glycosaminoglycans (sGAGs) was analyzed using the dimethylmethylene blue spectrophotometric method [51]. All samples were digested in papain solution (300 μg/ml in phosphate-buffered saline with 5 mM ethyl-enediaminetetraacetic acid and 5 mM cysteine; Sigma) at 65°C for 16 hours, then vortexed, and stored at -20°C. sGAG content was measured by mixing an aliquot (40 μl) of digested samples with dimethylmethylene blue dye solution (125 μl, 21 μg/ml, pH 3; two replicates per sample) in a 96-well assay plate and measuring absorbance (535 nm) on a plate reader (Tecan Genios, Mannendorf, Switzerland). sGAG concentrations were calculated from the absorbance using a standard curve prepared from commercial chondroitin-4-sulfate (Sigma). Total sGAG per sample was then normalized to DNA content (Quant-iT PicoGreen dsDNA Kit; Life Technologies) for each sample. Differences in sGAG production (sGAG/DNA) amongst control and treatment groups at each time point were evaluated via one-factor ANOVA, followed by Tukey’s *post-hoc* analysis at a significance level of 0.05 (*n* = 4).

### Sircol collagen assay

As described above, all papain-digested samples were thawed and vortexed prior to undergoing collagen content analysis according to the instruction of the Sircol Collagen Assay Kit (Biocolor, Carrickfergus, UK). Digested samples were incubated with the Sircol Dye reagent for 30 minutes, where each sample was gently mixed every 5 minutes. Samples were then centrifuged at 10,000×*g* for 10 minutes to collect the collagen-bound dye. Following supernatant removal, the pellet was resuspended in the Alkali reagent and transferred to a 96-well assay plate for measuring absorbance (535 nm) on a plate reader (Tecan Genios) with the total collagen content calculated using a standard curve. Two duplicate wells were measured for each sample, and each collagen content value was normalized to DNA content (as described above) for each sample. Differences in total collagen production (collagen/DNA) amongst control and treatment groups at each time point were evaluated via one-factor ANOVA, followed by Tukey’s *post-hoc* analysis at a significance level of 0.05 (*n* = 4).

## Results

### Expression of cell surface receptors in undifferentiated HUCMSCs

**Figure 1:**
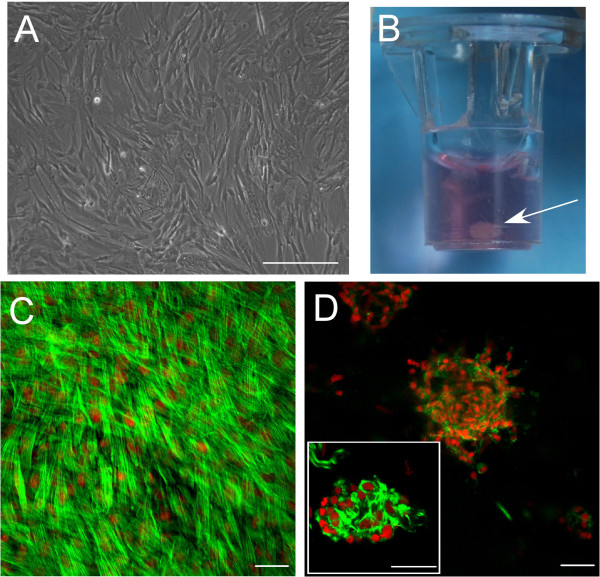
**Morphological features of undifferentiated/differentiated human umbilical cord mesenchymal stromal cells cultured on different surfaces. (A)** Undifferentiated cells were characterized by a fibroblast-like cell morphology during proliferation on a tissue culture polystyrene surface. **(B)** Differentiated cells formed a cell-clustering structure (arrow) in a Matrigel™-coated Transwell insert following culture in a pseudo-three-dimensional system for 21 days. **(C)** Differentiated cells showed morphology with stress fibers on a 0.1% gelatin solution-coated hard surface. **(D)** In comparison, differentiated cells formed a cluster structure on a 100% Matrigel™ soft surface (see high-magnification insert in **(D)**). **(C)**, **(D)** Cells were seeded on the wells of glass chamber slides precoated with 0.1% gelatin solution to improve cell attachment on the glass surface or 100% Matrigel™ to create a soft gel surface for cell clustering behavior respectively. They were cultured for up to 7 days, then fixed and stained for actin distribution by fluorescein isothiocyanate–phalloidin (green). Cell nuclei were counterstained with propidium iodide (red). Scale bars = 50 μM.

Undifferentiated HUCMSCs exhibited fibroblast-like morphology (Figure 1A) during proliferation on a tissue culture plastic surface. They expressed typical MSC markers, such as CD44, CD29, CD90, CD105 and CD166 (Table 2), but were negative for CD45, CD34 and CD31 (Table 2). Based on flow cytometry analysis, 98 to 99% of cells were positive for these MSC markers regardless of cell passage number (at passage 3 or passage 5), except for CD105 (75% positive cells at passage 3 and 25% at passage 5) (Table 2). Undifferentiated HUCMSCs also showed expression of many laminin–related cell surface receptors, such as Lutheran glycoprotein (CD239), integrin subunit α3 (CD49c) and α6 (CD49f), but not integrin β4 (CD104) (Table 2). Interestingly, limited passages (passage 3 to passage 5) did not affect cell surface expression for these receptors – integrin α6 being an exception. The expression of integrin α6 could not be detected in cells at passage 5 (Table 2). Only passage 3 cells were therefore used for the following cell differentiation study.

**Table 2:** Undifferentiated HUCMSCs express cell surface markers typical to MSCs and immature nucleus pulposus cells

	**Passage 3**		**Passage 5**	
**Target**	**% (+) cells**	**MFI**	**% (+) cells**	**MFI**
CD44	99	2758	99	917
CD90	99	366	99	120
CD166	98	216	99	53
CD105	75	16	26	9
CD29 (ITGβ1)	99	215	99	692
CD49c (ITGα3)	99	127	99	211
CD49f (ITGα6)	63	34	0	0
CD104 (ITGβ4)	1	12	2	2
CD239 (Lu)	30	17	82	32
CD34	0	0	0	0
CD31	0	0	1	1
CD45	0	0	0	0

Values represented a mean of duplicate samples (*n* = 2). %(+), percentage of total number of cells expressing the markers. HUCMSC, human umbilical cord mesenchymal stromal cell; ITG, integrin; Lu, Lutheran glycoprotein; MFI, mean fluorescence intensity; MSC, mesenchymal stem cell.

Data were obtained via flow cytometry analysis.

### Pseudo-three-dimensional culture of human umbilical cord mesenchymal stromal cells

#### Cell morphology in differentiation

A pseudo-three-dimensional culture condition (LM-111 rich, Matrigel™) promotes HUCMSC differentiation under no serum conditions. Between days 1 and 7 of culture on Matrigel™, HUCMSCs demonstrated a cell clustering morphology (Figure 1D) similar to that of immature NP cells *in situ*[52] and of primary immature NP cells within a similar laminin-rich culture system [47]. In comparison, HUCMSCs on a 0.1% gelatin solution-coated hard surface showed a flattened morphology with stress fibers (actin stained by fluorescein isothiocyanate–phalloidin) (Figure 1C). This suggests that the Matrigel™ culture system promoted HUCMSC clustering as we have observed with primary NP cells. By the end of 21 days, differentiated cells in control and two treatment groups (TGF-β1, IGF-1) had all formed gelatinous structures in the pseudo-three-dimensional laminin-rich culture system (Figure 1B).

#### Gene expression of nucleus pulposus markers

**Figure 2:**
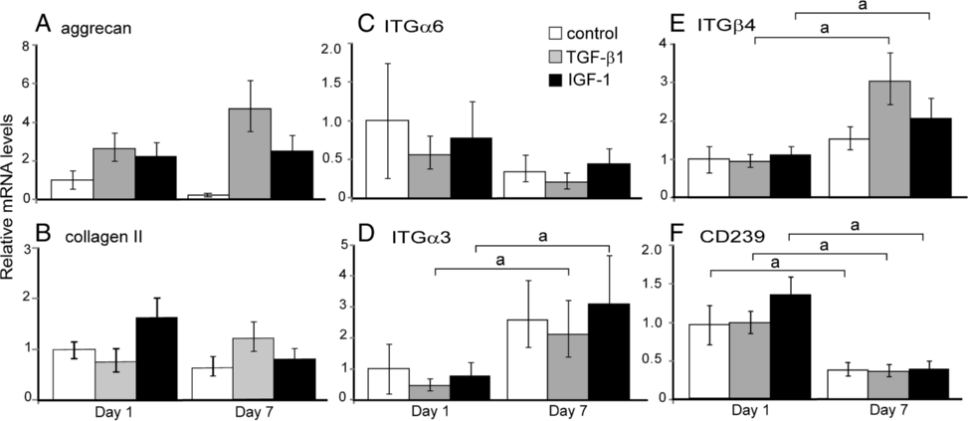
**Gene expression levels varied in human umbilical cord mesenchymal stromal cells during early differentiation.** Gene expression levels were measured via real-time RT-PCR. Relative mRNA fold-changes (2^–∆∆Ct^) for extracellular matrix proteins (**(A)** aggrecan, **(B)** type II collagen) and laminin receptors (integrin subunits: **(C)** ITGα6, **(D)** α3, **(E)** β4 and **(F)** CD239) in differentiated human umbilical cord mesenchymal stromal cells cultured on a three-dimensional Matrigel™ system for 1 and 7 days (*n* = 4, mean ± standard error). The relative mRNA level of the control sample at day 1 was set as the calibrator (value = 1) for normalizing the relative mRNA levels of treatment groups at day 1 and all groups at day 7 (a, fold-difference between day 1 and day 7 samples of each treatment group ≥2, *P* <0.05, one-factor analysis of variance followed by Tukey’s *post-hoc* analysis). IGF-1, insulin-like growth factor-1; TGF, transforming growth factor.

To monitor HUCMSC differentiation towards an NP-like phenotype, gene expression of NP-related ECM proteins and laminin receptors was analyzed via real-time PCR at days 1 and 7. Differentiated HUCMSCs expressed variable mRNA levels of NP markers at day 7 compared with that at day 1. For ECM proteins, mRNA levels of aggrecan appeared to increase from day 1 to 7 in the TGF-β1 treatment group (Figure 2A and Table 3), but the fold-increase was not considered statistically significant (Figure 2A and Table 3; *P* >0.05, ANOVA). No increase in mRNA levels was detected for type II collagen in any experimental group from day 1 to day 7 (Figure 2B). For integrin subunits, mRNA levels of integrins α3 and β4 increased from day 1 to day 7 for cells in all groups (Figure 2D,E), but only cells in both TGF-β1 and IGF-1 treatment groups exhibited statistically significant increases in integrins α3 and β4 (Figure 2D,E and Table 3; ≥2-fold, *P* <0.05, ANOVA). mRNA levels of CD239 and integrin α6 were decreased from day 1 to day 7 in all experimental groups (Figure 2C,F), with only the reduction in CD239 expression being statistically significant (Table 3 and Figure 2F; ≥2-fold, *P* <0.05, ANOVA).

**Table 3:** **∆C**_**t**_**values for target genes (target C**_**t**_**– β2**-**microglobulin C**_**t**_**)**

**Sample**	**Aggrecan**	**Collagen II**	**ITGα6**	**ITGα3**	**ITGβ4**	**CD239**
Control						
**Day 1**	**21.1 ± 0.7**	**17.6 ± 0.2**	**14.6 ± 1.0**	**8.7 ± 1.0**	**11.2 ± 0.5**	**13.3 ± 0.4**
Day 7	23.4 ± 0.3	18.3 ± 0.3	16.1 ± 0.9	7.4 ± 0.4	10.6 ± 0.3	14.7 ± 0.5^a^
TGF-β1						
Day 1	19.8 ± 0.4	18.0 ± 0.8	15.5 ± 0.2	9.9 ± 0.2	11.3 ± 0.2	13.3 ± 0.2
Day 7	18.9 ± 0.4	17.3 ± 0.7	16.9 ± 0.9	7.7 ± 0.5^a^	9.6 ± 0.4^a^	14.7 ± 0.5^a^
IGF-1						
Day 1	20.0 ± 0.5	16.8 ± 0.6	15.0 ± 0.9	9.1 ± 0.6	11.0 ± 0.2	12.8 ± 0.3
Day 7	19.8 ± 0.5	18.0 ± 0.7	15.8 ± 0.2	7.1 ± 0.4^a^	10.0 ± 0.4^a^	14.6 ± 0.5^a^

Values represent mean ± standard deviation of all RT-PCR replicates for *n* = 4 RNA samples. Control values (in bold) at day 1 are used to normalize all data as follows: ∆C_t_ values of control at day 1 are subtracted from ∆C_t_ values of control at day 7 or ∆C_t_ values of each treatment group at both day 1 and day 7 to obtain ∆∆Ct and the fold-difference of relative mRNA levels (2^–∆∆Ct^) for each target as illustrated in Figure 2. IGF-1, insulin-like growth factor-1; ITG, integrin; TGF, transforming growth factor.

^a^Fold-difference between day 1 and day 7 samples ≥2 and *P* <0.05, one-factor analysis of variance followed by Tukey’s *post-hoc* analysis.

#### Glycosaminoglycan and collagen synthesis

**Figure 3:**
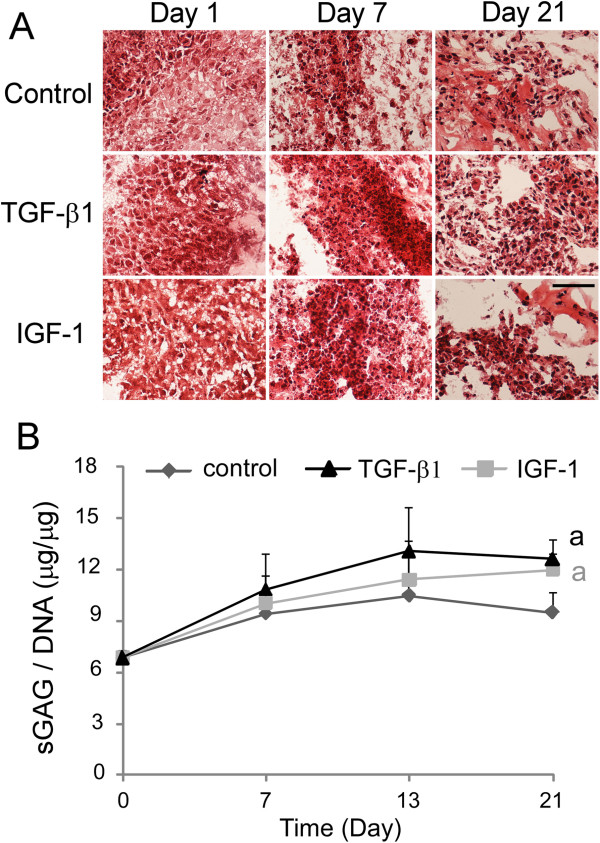
**Glycosaminoglycan production detected in the differentiated human umbilical cord mesenchymal stromal cells cultured in Matrigel™.** Human umbilical cord mesenchymal stromal cells were cultured for 1 to 21 days in a three-dimensional Matrigel™ system. **(A)** Histological staining for proteoglycan by safranin O (*n* = 4, only one represented image shown; scale bar = 50 μm). **(B)** Sulfated glycosaminoglycan production normalized to DNA content (sGAG/DNA, *n* = 4, mean ± standard error) in cells (a, significant difference was detected between either treatment groups and control at day 21 only, one-factor analysis of variance, followed by Tukey’s *post-hoc* analysis, *P* < 0.05). IGF-1, insulin-like growth factor-1; TGF, transforming growth factor.

Safranin-O staining and sGAG production were maintained in all cells cultured up to 21 days (Figure 3). No difference in safranin-O staining was observed amongst two growth factor treatment groups and control group at any time point (Figure 3A). By the end of day 21, however, both TGF-β1 and IGF-1 treatment groups exhibited significantly elevated levels of total sGAG/DNA, as compared with that in the control group (Figure 3B; *P* <0.05, ANOVA).

**Figure 4:**
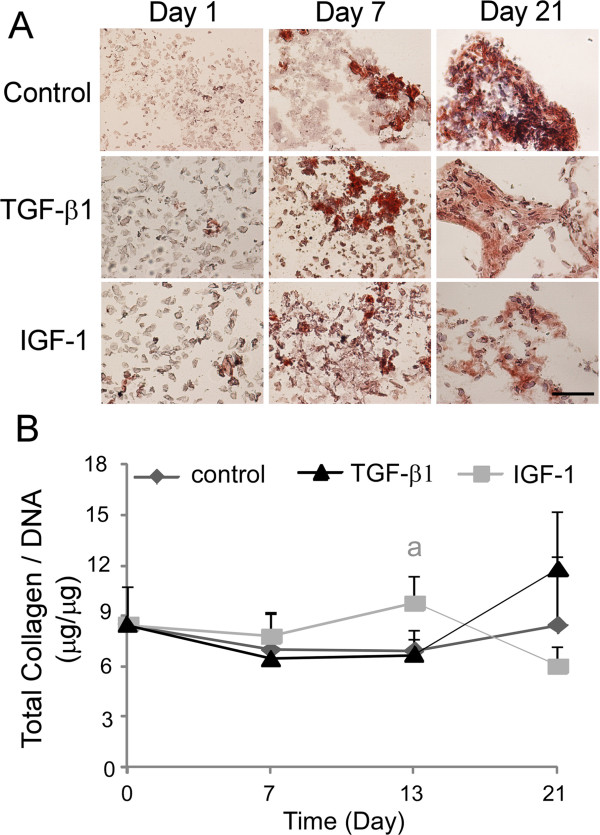
**Total collagen production and type-II collagen protein in the differentiated human umbilical cord mesenchymal stromal cells cultured in Matrigel™.** Human umbilical cord mesenchymal stromal cells were cultured for 1 to 21 days in a three-dimensional Matrigel™ system. **(A)** Immunostaining for type II collagen (Col II) (*n* = 4, only the represented image shown; scale bar = 50 μm). **(B)** Total collagen content normalized to DNA (collagen/DNA, *n* = 4, mean ± standard error) in cells (a, significant difference was detected between IGF-1 treatment group and control at day 13 only, one-factor analysis of variance, followed by Tukey’s *post-hoc* analysis, *P* <0.05). IGF-1, insulin-like growth factor-1; TGF, transforming growth factor.

Differentiated HUCMSCs from all experimental groups stained intensely for type II collagen at day 21 (Figure 4A). An increase of staining intensity was observed across all treatment groups and control from day 7 to day 21, but almost no positive staining appeared at day 1 for all groups (Figure 4A). Total collagen/DNA contents were maintained in all cells cultured up to 21 days (Figure 4B). There was no difference for total collagen/DNA contents observed amongst two growth factor treatment groups and control at day 7 and day 21 (Figure 4B). However, at day 13 cells from the IGF-1 treatment group showed significant increases in total collagen/DNA content relative to the control and TGF-β1 treatment group (Figure 4B; *P* <0.05, ANOVA).

#### Protein expression of nucleus pulposus markers

**Figure 5:**
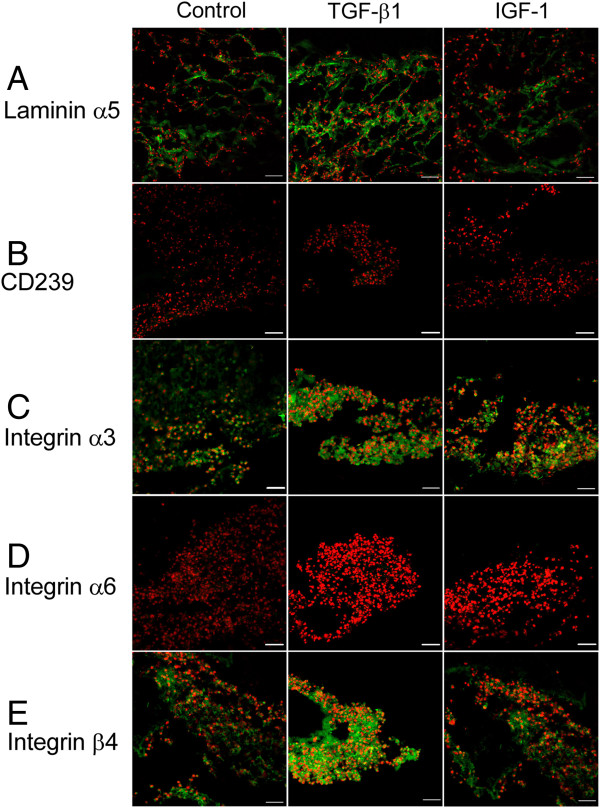
**Differentiated human umbilical cord mesenchymal stromal cells expressed nucleus pulposus cell markers.** Immunostaining for **(A)** the laminin (LM) α5 chain and receptors, **(B)** CD239, **(C)** integrin subunit α3, **(D)** integrin subunit α6 and **(E)** integrin subunitβ4 in differentiated human umbilical cord mesenchymal stromal cells cultured for 21 days (green = target; red = cell nuclei counterstained with propidium iodide; bar = 50 μm). IGF-1, insulin-like growth factor-1; TGF, transforming growth factor.

After 21 days in culture, differentiated HUCMSCs from all experimental groups stained positively for the laminin α5 chain (Figure 5A), although protein expression of the laminin α5 receptor (CD239) was not detected in any group (Figure 5B). Protein expression of integrins in differentiated HUCMSCs was maintained for both α3 and β4 subunits (Figure 5C,E) but no expression of the α6 subunit was detected in any group (Figure 5D). No difference in protein expression levels for these NP markers was observed amongst the two growth factor treatment groups and control (Figure 5).

## Discussion

The goal of this research was to explore suitable environmental conditions to promote the differentiation of HUCMSCs into cells with an immature NP-like phenotype. These conditions were designed by growing cells in a pseudo-three-dimensional environment using Matrigel™ as a laminin-rich extracellular matrix environment alone or with the addition of the growth factors IGF-1 and TGF-β_1_, and using hypoxic conditions. This study demonstrated that HUCMSCs have some potential to differentiate into cells sharing features with immature NP cells within a laminin-rich pseudo-three-dimensional culture environment. Our results indicated that HUCMSCs not only formed matrix similar to that of NP cells, but also expressed some immature NP cell markers during differentiation. Extracellular matrix protein (collagen II, laminin α5), glycosaminoglycans (sGAGs by both safranin-O staining and dimethylmethylene blue assay) and some laminin receptor protein expression (integrin α3 and β4 subunit) were detected in differentiated cells under all culture conditions. These expression patterns of the laminin isoform and its receptors are similar to that in immature NP tissue *in situ*, as we reported previously [45]. These finding suggest that the soft Matrigel™ culture system was directly responsible for regulating HUCMSC differentiation here. As early as 24 hours post seeding, these cells were shown to cluster in a manner akin to that of both immature NP cells *in situ* and primary immature NP cells cultured in a similar laminin-rich culture system [47]. These observations suggest that the laminin-rich pseudo-three-dimensional culture system here provided HUCMSCs with a suitable microenvironment similar to that of the native NP tissue including the presence of free laminin ligand and soft mechanical properties for the substrate.

ECM interaction with cells is well known to play an important role in stem cell differentiation [53]. Previously, laminin was found to enhance adult MSC differentiation to adipocytes [54] and human embryonic stem cell differentiation into neurons [55]. It is also known that substrate stiffness affects the fate of stem cell differentiation [56]. Our previous study revealed that mechanical properties of Matrigel™ are in a range spanning that of the gelatinous-like NP tissue. Indeed, immature NP primary cells maintained a clustered morphology when they were cultured on this Matrigel™ substrate [47]. We believe that this soft substrate also promotes and maintains clustered cell morphology during HUCMSC differentiation in the current study. Nevertheless, our results highlighted the effect of both laminin ECM proteins and substrate stiffness on HUCMSCs differentiation into immature NP-like cells *in vitro*.

It is noteworthy that not all NP-associated laminin receptors were maintained in differentiated HUCMSCs at the end of the culture time (21 days). The integrin α6 subunit that forms a complex with the β4 subunit and CD239, a laminin α5 subunit-specific receptor, were unexpectedly absent in differentiated cells in the control group and two treatment groups. However, LM-511 and its receptors (integrin α3, integrin β4) were highly expressed in all differentiated cells at day 21 (Figure 5). The differential expression of receptor types at the protein levels appeared to be reflected in their mRNA levels during differentiation. It was noted that the gene expression levels of integrins α3 and β4 were elevated while the gene expression levels of integrin α6 and CD239 were decreased from day 1 to day 7 for cells in all groups. It is possible that LM-111, the major component of Matrigel™, only promotes the expression of integrins receptors but not the non-integrin receptor, CD239. In a future study, a LM-511 supplement may be required to promote expression of the specific receptor CD239 in differentiated cells.

Growth factors provide molecular signaling and cues that promote cells to differentiate along specific pathways from an undifferentiated state to a differentiated state. Two growth factors that seem particularly important for differentiation towards IVD-like cells are IGF-1 and TGF-β1. IGF-1 and its receptor have been shown to be expressed in the IVD [57,58], and IGF-1 has been shown to have an effect on early dorso-anterior (notochord) development [59] and to promote chondrocyte differentiation and embryonic bone development [60]. Members of the TGF-β family and receptors are expressed in the IVD [57,58], and TGF-β1 was reported to be able to induce rat MSCs to differentiation to a phenotype consistent with NP on alginate hydrogels [19]. However, supplementation with either IGF-1 (500 ng/ml) or TGF-β1 (1 ng/ml) did not generate distinct differences in NP-like cell types in the current study; although sGAG content was enhanced at day 21 of culture in both growth factor treatment groups relative to the control of Matrigel™ alone. Similarly, total collagen content was significantly elevated at day 13 in IGF-1 treated group. Furthermore, we selected a special form of Matrigel™ with relative lower growth factor residuals (BD Biosciences) for this study to minimize the possible interference from the endogenous growth factors in Matrigel™. Together, our finding may imply that the laminin matrix niche, substrate softness and three-dimensional culture, but not growth factors, are more critical factors in stimulating HUCMSC differentiation toward an immature NP-like phenotype in the Matrigel™ system. Future studies with various concentrations of growth factor supplements and using different ECM matrixes (such as collagen or agarose) as well as no matrix as possible controls are needed to confirm these new findings. In addition, a larger sample size may be needed to statistically reveal a greater benefit of using growth factors.

Although the differentiated HUCMSCs developed a shape similar to that of the NP cells, the final tissue constructs (cell clusters) were quite small. Larger cell clusters could be made by increasing the number of HUCMSCs during initial seeding with some important benefits. First, cells could be differentiated into a cell structure that truly resembles the NP and that could potentially replace damaged NP in the future. Second, larger cell clusters could be used to analyze the mechanical properties of the HUCMSC cell cluster. In preliminary tests, we attempted to measure shear stress using a rheometer. Unfortunately, the cell clusters were too small to measure significant values for shear stiffness in bulk. Larger cell clusters would allow for the measurement of these properties. The mechanical properties of these constructs will provide quantitative data to test whether formed HUCMSC clusters are adopting a gelatinous immature NP-like phenotype.

## Conclusions

Our study demonstrates that HUCMSCs have the potential to differentiate into cells sharing features with immature NP cells within a laminin-rich pseudo-three-dimensional culture system. Differentiated HUCMSCs not only formed a gelatinous cell clustering structure and produced matrix proteins (collagen II, laminin α5) and sGAGs similar to that of immature NP cells, but also expressed some immature NP cell markers (laminin receptors: integrin α3 and β4 subunit). Growth factor treatments had no effect on the expressions of immature NP markers in the differentiated HUCMSCs.

## Abbreviations

ANOVA: Analysis of variance; CD: Cluster of differentiation (cell surface molecule); ECM: Extracellular matrix; HUCMSC: Human umbilical cord mesenchymal stromal cell; IGF: Insulin-like growth factor; ITG: Integrin; IVD: Intervertebral disc; LM: Laminin; MSC: Mesenchymal stem cell; NP: Nucleus pulposus; sGAG: Sulfated glycosaminoglycan; TGF: Transforming growth factor.

## Competing interests

The authors declare that they have no competing interests.

## Authors’ contributions

JC, BHC and LAS conceived and designed the experiments. BHC, EJL and LJ performed the experiments. BHC, EJL, LJ and LAS analyzed the data. EJL, JC and LAS wrote the paper. All authors edited and approved the manuscript for publication.
